# Brightening the Day With Flashes of Positive Mental Imagery: A Case Study of an Individual With Depression

**DOI:** 10.1002/jclp.22455

**Published:** 2017-02-02

**Authors:** Simon E. Blackwell, Emily A. Holmes

**Affiliations:** ^1^ Ruhr‐Universität Bochum; ^2^ Karolinska Institutet; ^3^ Medical Research Council Cognition and Brain Sciences Unit

**Keywords:** mental imagery, depression, anhedonia, involuntary memories, cognitive bias modification

## Abstract

This article presents a case example of an individual with current major depression engaging in a positive mental imagery intervention, specifically a computerized cognitive training paradigm involving repeated practice in generating positive imagery in response to ambiguous scenarios. The patient's reported experience of the intervention suggests the potential of the positive imagery intervention to “brighten” everyday life via promoting involuntary “flashes” of positive mental imagery in situations related to the scenarios, with associated beneficial effects on positive affect, future expectations, and behavior. Enhancing this aspect of the training–i.e., involuntary positive imagery in contexts where it is adaptive–may hold particular promise for reducing anhedonic symptoms of depression. Developing simple computerized interventions to increase the experience of positive mental imagery in everyday life could therefore provide a useful addition to the drive to improve treatment outcomes.

This article uses the case of an individual with depression to illustrate how a computerized positive mental imagery training program might help someone experience more positive emotion, imagine more positive events in the future, and regain interest and enjoyment in everyday life. Could a simple computer program really provide significant benefits and even tackle aspects of depression, such as anhedonia, that pose a particular challenge to current treatments? This article explores this idea via the example of “Theresa,” a 50‐year‐old woman with major depression, and suggests directions for future treatment research to work towards this possibility.

Depression presents such a global health problem not only because it is prevalent and disabling, but also because of the increasingly apparent limitations of existing treatment approaches, whether psychological or pharmacological. Many people struggle to access treatment, and of those who do, a substantial proportion will either not respond or respond but then quickly relapse (Bockting, Hollon, Jarrett, Kuyken, & Dobson, 2015). While increasing the efficacy and accessibility of existing treatments will yield benefits, the prevalence of the disorder means that new kinds of treatment approaches that can be easily and efficiently “scaled up” are needed (cf. Kazdin & Blase, 2011). One way to develop more efficient and scalable treatments may be via identifying mechanisms involved in the maintenance of a disorder and developing more direct ways to target these (Holmes, Craske, & Graybiel, 2014).

According to cognitive theories, depression is maintained by a number of cognitive biases, dysfunctional patterns in processes such as interpretation, attention, and memory (Gotlib & Joormann, 2010), and thus these may present useful targets for interventions. Evidence from experimental psychopathology research suggests that such biases are amenable to modification via simple computerized cognitive training procedures, commonly referred to cognitive bias modification (CBM). There has been increasing interest in the potential of such procedures to re‐train dysfunctional cognitive biases and thus reduce symptoms of psychopathology (Woud & Becker, 2014).

One line of research in depression has started to investigate a CBM paradigm focussing on two cognitive targets: mental imagery and interpretation. Depression is characterized by impoverished positive mental imagery (Holmes, Blackwell, Burnett Heyes, Renner, & Raes, 2016) and bias to interpret ambiguous information negatively (Rude, Wenzlaff, Gibbs, Vane, & Whitney, 2002). In “positive imagery CBM,” participants repeatedly practice imagining positive resolutions for ambiguous training stimuli, with the aim of training a more positive bias to imagine positive resolutions for ambiguity in everyday life. Initial experimental studies in nonclinical samples established that a single session induces a more positive interpretive bias, increases positive affect, and acts as a “cognitive vaccine” to protect against mood deterioration (e.g. Holmes, Lang, & Shah, 2009).

Subsequent preliminary studies in clinical samples, adults with current major depression, found promising results from a one‐week training schedule completed from home in reducing symptoms of depression and negative interpretation bias (e.g. Blackwell & Holmes, 2010). In a subsequent randomized controlled trial (RCT), 150 adults with current major depression were randomized to complete either a four‐week positive imagery CBM schedule via the internet or a “sham training” control condition (Blackwell et al., 2015). However, there was no difference between the two conditions in the trial's primary outcome measure, reduction in symptoms of depression.

While the unexpected results of Blackwell et al. (2015) highlighted some of the potential difficulties that may be encountered in the process of clinical translation, they also suggested areas of promise for future research. Exploratory analyses of the trial's “per protocol” sample (i.e., those participants who completed a threshold number of sessions and provided outcome data), found that participants in the positive imagery condition reported a greater reduction in anhedonic symptoms of depression than those in the control condition (Blackwell et al., 2015).

This finding is particularly interesting because anhedonia, the loss of interest in or pleasure from previously enjoyed activities, responds poorly to current treatment approaches (Dunn, 2012). While subsequent studies using a one‐week training schedule further indicate the potential of positive imagery CBM to reduce anhedonia (Pictet, Jermann, & Ceschi, 2016; Williams et al., 2015), further work is needed to confirm and enhance this potential effect.

In summary, positive imagery CBM is in the early stages of research as a potential treatment tool in depression. A brief, simple computerized cognitive training procedure that reduced symptoms of depression, and anhedonic symptoms in particular, could provide an easily accessible and scalable intervention or treatment adjunct. However, while research to date indicates potential promise, it also suggests a need for further development. Identifying ways to enhance the effects of training could be especially useful. In the context of treatment development, examination of individual cases can provide valuable information to suggest ways forwards, by generating hypotheses for future research or challenging potential assumptions (Kazdin, 2014). For example, examination of individual cases can provide unexpected insights about potential conditions or mechanisms of treatment success or failure that may be lost when averaged across a study sample. The hypotheses generated can then be tested in future experimental studies or clinical trials.

The case study that follows describes the experience of one participant who took part in the RCT of Blackwell et al. (2015) and showed marked improvements. It is chosen as an example that illustrates some interesting processes that may contribute to the beneficial effects of a positive imagery CBM intervention, suggesting hypotheses for future research and clinical application. After the client description, an initial formulation is presented to explain how the positive imagery CBM would be hypothesised to reduce symptoms of depression. After the description of the treatment process and outcomes, an updated formulation is proposed to accommodate additional potential treatment mechanisms highlighted by the client's account of her experience. The implications for research and clinical applications are then discussed.

## Case Illustration

### Presenting Problem and Client Description

“Theresa” was a widowed, White British woman in her 50s who saw an advertisement for the study in her local newspaper. Her doctor had suggested that she was depressed 2 years previously and had recommended antidepressants, but she had declined these because she felt that medication would not “solve the problem” of why she was depressed. Theresa had few expectations for the online computer program she had seen advertised but was interested to take part in the study as an opportunity to “do something useful.”

During the study eligibility assessment, Theresa reported a long history of recurrent depression since the age of 21, and estimated her current major depressive episode to be of 4 years duration. The diagnostic interview revealed a number of comorbidities, namely, current posttraumatic stress disorder (from a physical assault at work 10 years previously); social phobia (since her late teens), which was characterized by a fear of coming across as “stupid” in conversations; a specific travel phobia (as long as she could remember), originally limited to public transport but which had recently spread to include driving her car; and generalized anxiety disorder (since her early teens), which was characterized by worry (especially in the evening or at night) about a range of topics from her own health to the life choices of her daughters. Theresa also had a history of alcohol dependence, and although she did not currently meet diagnostic criteria, she felt that she was drinking more than she should do. Theresa was currently receiving no treatment for her depression or anxiety. She was currently taking codeine for chronic pain, which she described as diffuse in nature, affecting many of her muscles and joints.

Despite her long history of depression and anxiety, Theresa had received little treatment in the past. She had taken Valium briefly in her early 20s and an antidepressant for a few years in her 30s. She had received a few sessions of counseling 5 years previously but had not found it helpful because she did not get on well with the counselor.

Theresa had held a number of administrative jobs in the past, but recently she had been reducing her hours and was increasingly worried about debt. She lived alone with her mother, who was in the early stages of Alzheimer's disease, and for whom she was the main caregiver. Theresa's husband had died 15 years previously, following a stroke; and although she had two adult daughters, they did not live locally and she saw them only infrequently.

On the baseline questionnaire assessment measures (see Table 1), Theresa scored in the “severe” range for depression on the Beck Depression Inventory – II (BDI‐II; Beck, Steer, & Brown, 1996), including anhedonic symptoms (rating “I get very little pleasure from the things I used to enjoy” and “I am less interested in other people or things than before”). On the Prospective Imagery Test (PIT; Holmes, Lang, Moulds, & Steele, 2008), she rated the vividness with which she could imagine positive events in her future as “vague and dim” on average. She scored nearly at ceiling on a measure of negative interpretation bias, the Scrambled Sentences Test (SST; Rude et al., 2002). Her score on the Behavioural Activation for Depression Scale (BADS; Kanter, Mulick, Busch, Berlin, & Martell, 2007) indicated very low levels of behavioral activation, consistent with her report that she was “not doing very much around the house” and that she often struggled to go out even to carry out simple errands. See Table 1 for exact scores on the measures and more information on how these are interpreted.

**Table 1:** Outcome Measures Over the Positive Imagery Intervention and Follow‐Up Period

	Baseline	Posttreatment	1‐month follow‐up	3‐month follow‐up	6‐month follow‐up
BDI‐II	39	11	5	6	25
Anhedonia	3	1	0	0	2
PIT vividness	2	2.8	3.6	3.9	2.3
SST negativity	.89	.58	−	−	−
BADS	61	75	134	134	88

*Note*. BDI‐II = Beck Depression Inventory Second Edition (range: 0 ‐ 63. Cut‐offs: 0‐13, minimal depression; 14‐19, mild depression; 20‐28, moderate depression; 29+, severe depression); PIT = Prospective Imagery Test, vividness ratings for positive scenarios (range: 1 = *no image at all* to 5 = *very vivid*); SST negativity = Scrambled Sentences Test negativity score (number of sentences completed negatively divided by total number completed correctly; range 0 to 1, where higher scores indicate a more negative bias); BADS = Behavioural Activation for Depression Scale (range: 0 to 150, where higher scores indicate greater levels of activation).

### Initial Case Formulation

Theresa presented with a long history of recurrent depression and numerous anxiety disorder comorbidities, suggesting longstanding vulnerability that may present a challenge to a brief intervention. Thus, while the formulation that follows focuses on cognitive processes, these are only one part of a clearly complex picture that includes a number of socioenvironmental stressors that could easily contribute to maintaining depressed mood.

Within the cognitive framework that had informed the investigation of positive imagery CBM, two processes can be identified that could contribute to the maintenance of Theresa's depression and be targeted by the intervention. First, Theresa demonstrated difficulty in imagining positive events in her future (as indicated by a low score on the PIT). Imagining future events is thought to contribute to individuals’ estimate of the likelihood of those events occurring, and their evaluation of the potential outcomes of a course of action. Thus, being unable to generate positive mental images (which in itself may be exacerbated by or be a consequence of depressed mood) may contribute to a sense of hopelessness about the future (i.e., low likelihood of positive events occurring) and reduce motivation to engage in potentially rewarding activities (i.e., unable to imagine that they might have positive outcomes), reinforcing depressed mood and behavioural withdrawal (Holmes et al., 2016). For example, if Theresa was to receive an invitation to a social event, she may be unable to imagine positive possible aspects of attending the event, such as enjoying herself or the other guest being very friendly.

Second, Theresa demonstrated a tendency to interpret ambiguous information negatively (as indicated by a high score on the SST). Given the inherently ambiguous nature of many of the situations encountered in everyday life, habitually interpreting these in a negative manner, rather than positively or benignly, would also reinforce depressed mood. For example, Theresa may interpret the invitation as meaning “They're just being polite; they don't really want me to come” rather than “They'd like to see me.”

Within such a cognitive framework, if a positive imagery CBM intervention could increase Theresa's ability to generate vivid positive mental imagery and train a more positive interpretation bias, then this could help reduce her symptoms of depression by enabling her to imagine positive possibilities in her future, and by increasing the chance that she may interpret everyday events in a positive or benign, rather than negative, manner.

### Course of Treatment

#### Positive imagery intervention

The positive imagery CBM intervention as implemented in the trial (Blackwell et al., 2015) comprised an initial face‐to‐face introductory session at the research center followed by 12 sessions completed from home over the Internet. Six sessions used audio presentation of training stimuli, in which participants listened to brief descriptions of everyday situations that were structured so that they started ambiguous as to their potential resolution but always ended positively (for example, “As you are getting dressed in the morning, you think about the day ahead. You anticipate it with *pleasure and feel full of energy*”; positive resolution in italics). Participants were instructed to imagine themselves in each scenario as if actively involved, seeing it through their own eyes, and in particular to focus on the scenario's outcome.

**Figure 1:**
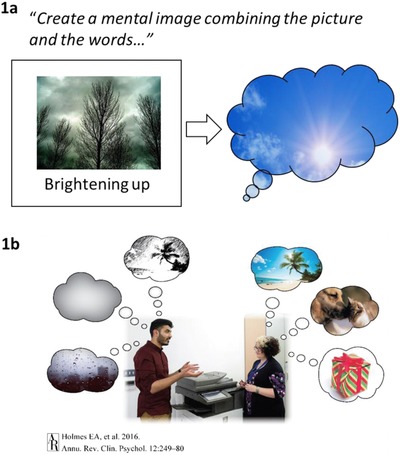
Illustration of (a) an example training stimulus from one of the computerized positive imagery training tasks; (b) the lack of positive mental imagery that characterizes depression. In one version of the training task, participants view a series of ambiguous photographs paired with a positive caption. Their task is to generate positive mental image that combines the photo with the caption. In this example (1a), the participant might imagine the clouds clearing and enjoying a sunny day. The possibility suggested by the case illustrated in this article is that the positive images generated during the training may “pop back” or “flash” to mind during their everyday life, so that the individual's experience becomes more like the person on the right‐hand side of (1b) rather than the person on the left. Figure 1b is reproduced with permission from the Annual Review of Clinical Psychology, Volume 12 © 2016 by Annual Reviews, http://www.annualreviews.org

In the other six sessions, training stimuli were ambiguous photographs of mostly everyday scenes, paired with a positive caption of a few words. Participants were instructed to generate a positive mental image that combined the photo and the caption. For example, if presented with a photo showing a cloudy grey sky combined with the caption “brightening up,” the participant may generate an image of the clouds clearing and enjoying a sunny day (see Figure 1). The training schedule alternated between the audio and picture‐word sessions, with six sessions (one each day) scheduled for the first week, and two sessions (one of each type) scheduled for each of the following 3 weeks. Each session comprised 64 training stimuli, arranged into eight blocks of eight scenarios, with a brief self‐paced break in between each block (approximately 20 minutes per session), and no stimulus was repeated over the course of the intervention.

As in the preceding experimental (Holmes et al., 2009) and clinical studies (Blackwell & Holmes, 2010), the face‐to‐face introductory session started with a standardized introduction to the imagery CBM program, guided by the researcher following a written protocol (available on request from the first author). This included an introduction to mental imagery and practice in generating mental imagery in response to scripts read aloud by the researcher. The introduction aimed to enhance the extent to which participants could feel actively emotionally involved in the mental imagery, for example, via emphasizing the generation of field (i.e., through one's own eyes) as opposed to observer (i.e., viewing oneself from the outside) perspective imagery, and via instructing participants to try to avoid verbally analysing the scenarios (cf. Holmes et al., 2009).

In addition, in order to reduce the risk that the consistently positive nature of the scenarios might lead to participants rejecting them as unrealistic, the fact that the scenarios might sometimes seem unrealistic was explicitly addressed. Participants practiced imagining obviously unrealistic situations, such as winning the lottery or seeing an elephant flying past through the clouds, to illustrate the point that in generating mental imagery, the realism or otherwise of a situation need not be an issue. Once participants had moved to the computer to start the training session, the researcher then sought feedback after each block of eight scenarios. This provided an opportunity to ascertain that participants understood the task instructions and to facilitate their engagement in the subsequent computerized training session.

There was a face‐to‐face outcome assessment at posttreatment (i.e. 4 weeks post‐baseline), and follow‐up measures were then completed online 1, 3, and 6 months after the end of the intervention. A final feedback interview, including debriefing, was carried out by telephone following completion of the 6‐month follow‐up questionnaires.

#### Progress through intervention

In the face‐to‐face introductory session, Theresa engaged well with the imagery CBM program, although she initially struggled with generating images while listening to audio scenarios. Via the researcher‐guided introduction to mental imagery and feedback elicited during breaks (see previous section), the researcher was able to identify that Theresa was struggling and provided suggestions to help her engage in the imagery. This included normalizing the difficulty Theresa was experiencing, reassuring her that the task was in fact challenging and it was thus not surprising she did not find it straightforward. The aim here was to avoid Theresa feeling like a “failure” or becoming disheartened by her difficulty, but rather to see it as normal part of the process and something that could be overcome. Further, examples of where Theresa had been able to generate a more vivid image were elicited and discussed. A strategy developed with Theresa was to stop trying to generate a detailed visual image of every aspect of the scenario as it unfolded, but rather to see the first section of the scenario as “setting the scene” and focus her efforts on more vividly imagining the (positive) outcome of the scenario and the feeling involved in the outcome.

Theresa completed all 12 online sessions and noticed her mood improving over the course of the 4‐week intervention. She felt that she became gradually better at generating positive imagery during the training, and she found herself thinking more positively about events in her life, consistent with the idea of increasing ability to generate positive mental imagery and the induction of a more positive interpretation bias.

#### Flashes of positive imagery in everyday life

In addition, Theresa reported some phenomena that did not appear to be accounted for in the initial conceptualization of the imagery CBM intervention as described in the “Initial Case Formulation.” She noticed that generating positive imagery during the sessions provided an immediate (and very welcome) boost to her mood. Intriguingly, this effect seemed to spread into her daily life via the positive images “*popping back*” into her mind, for example, if triggered by encountering a situation that was similar to one of the training scenarios. The image would not simply be a visual scene, but included some of the positive emotion generated by imagining it during the session, and some of the associated cognition, such as expectation of a positive outcome.

Theresa reported that sometimes such an involuntary memory of a training scenario could influence her behavior. She provided a particularly striking example of a training scenario that had involved going for a walk and feeling invigorated. On more than one occasion, this image had popped into her mind, bringing with it a burst of positive emotion (experienced as anticipatory pleasure) and prompting her to then go out for a walk. Noticing the beneficial impact that the positive imagery had on her mood and general outlook prompted Theresa to bring some of the positive images generated during training sessions deliberately to mind during daily life to provide a boost to her mood.

### Outcome and Prognosis

Theresa's scores on the posttreatment outcome measures (Table 1) showed marked improvement, including a reduction in symptoms of depression into the “minimal” range, a lower negativity score on the SST, and improved ability to imagine positive events. She felt that the program had helped her “tremendously” and, in fact, wished that she could continue using it (not possible within the constraints of the trial). Theresa reported general increases in behavioral and social engagement–for example, she had made inquiries about joining a local choir and looked into membership of a gym–and this improvement is reflected in increased score on the BADS (Table 1). Theresa reported that others had also noticed the improvements. For example, one of her daughters had commented on how well she looked, which had made her notice that she was starting to care about her appearance again, for example, in how she dressed and her use of make‐up. At a hospital appointment, where normally she would be very anxious, a nurse had commented on how calm she seemed. Theresa had also stopped drinking alcohol completely and was pleased and surprised that this had been possible.

Theresa maintained her improvement over the following 3 months of follow‐up, with no anhedonic symptoms and high levels of behavioral activation (Table 1). Shortly before the final 6‐month follow‐up, she experienced some worsening of her symptoms, which she attributed to a difficult family situation. However, she felt that she was coping much better than she would have done previously and was pleased because she had maintained her abstinence from alcohol. Theresa reported that she still brought to mind some of the positive images to help improve her mood, and this also helped to stop her from “hitting the bottle.”

In her feedback, Theresa was very positive about the imagery CBM intervention, rating herself as “extremely satisfied” and “extremely confident” in recommending it to a friend with depression (in fact, she commented that she had already done so). She rated herself as “extremely likely” to be willing to try the program again if depressed in the future and said that she wished she had access to it at the current time because she thought it would be beneficial.

## Clinical Practices and Summary

### Reformulation of Treatment Effect: Flashes of Positive Imagery in Everyday Life

Theresa's account of her improvement over the course of the positive imagery CBM intervention suggests potential mechanisms for the beneficial effects of the intervention that are not accounted for in the theoretical accounts initially motivating the investigation of positive imagery CBM in depression. As described in the “Initial Case Formulation” section above, according to this initial conceptualization, it would be the induction of a more positive interpretation bias and increased vividness with which Theresa could imagine positive events in her future that would drive the reduction in her depression symptoms. While Theresa's reduced score on the SST and increased score on the PIT are consistent with such an account, her description of the process by which her symptoms improved suggests some additional, intriguing possibilities.

Theresa noticed and experienced as beneficial the positive affect directly induced as a result of generating positive imagery during the sessions themselves; for someone who is depressed, anhedonic, and experiences little positive affect, even transient boosts may be welcome. While increases in positive affect over a session of imagery CBM have been demonstrated in experimental studies with healthy volunteers (e.g. Holmes et al., 2009), this phenomenon has received little attention in clinical studies.

**Figure 2:**
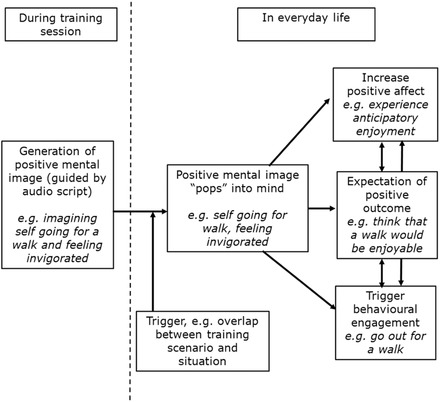
Illustration of one route via which a computer‐based positive mental imagery training program could have beneficial effects in depression, via reexperiencing of the images generated during training in everyday life “popping” or “flashing” into mind. Associated increases in positive affect, expectation of positive outcomes, and behavioural engagement could lead improvements in depression over time via, for example, reductions in anhedonia (loss of interest in and enjoyment from activities) and increases in behavioural activation.

Particularly striking however was how the benefits of generating positive imagery appeared to extend beyond the training session themselves, via reexperiencing of the images in daily life (e.g., having an image of going for a walk and finding it invigorating popping into her mind). Both involuntary and voluntary recall of the positive images brought with them the associated positive affect (e.g., anticipatory pleasure of going for the walk) positive future‐oriented cognition (e.g., the thought that going for a walk would be an enjoyable activity) and could also influence behavior (e.g., actually going out for a walk). Thus, increased experience of positive mental images in daily life, specifically those generated during training sessions, appeared to lead to increased experiences of positive affect, increased positive future‐oriented cognitions (i.e., expectation of positive outcomes), and increased behavioral activation, which together could contribute to reducing symptoms of depression (see Figure 2).

Increasing the experience of positive affect in daily life and the expectation of enjoyment from activities may have particular benefits for reducing anhedonic aspects of depression, consistent with the findings of Blackwell et al. (2015), and the reduction to zero of Theresa's anhedonic symptoms. Further, the hypothesised link between the positive imagery and behavioral activation fits with the observation that participants in the positive imagery condition in the trial showed initially greater increases in self‐reported behavioral activation (as measured by the BADS) than those in the control condition (Renner, Ji, Pictet, Holmes, & Blackwell, 2016). While such a formulation is of course speculative, it has interesting implications for the development and application of positive imagery CBM as a treatment tool.

### Implications for Research

Theresa's case, highlighted by her own reports, suggests that research into positive imagery CBM could benefit from investigating additional potential mechanisms of change. The proposal that the generation of positive mental images during a session of positive imagery CBM may lead to increased experience of positive mental imagery in daily life via involuntary retrieval of those images generated during a session (Figure 2) requires formal investigation.

Such a possibility would have implications for how best to optimize the training to increase the likelihood of such involuntary retrieval (cf. Berntsen, Staugaard, & Sørensen, 2013). For example, increasing the positive affect generated while imagining a scenario during the training may increase the chance that the image later returns as a positive involuntary memory (e.g. Clark, Mackay, & Holmes, 2013). The involuntary positive images were not simply “pictures” in her mind, but appeared to be associated with the positive affect, positive future expectancies (cf. Blackwell et al., 2013; Ji, Holmes, & Blackwell, 2017), and potential behavioral courses of action simulated while generating the image during training (cf. bioinformational accounts of mental imagery; Ji, Burnett Heyes, MacLeod, & Holmes, 2016); these aspects of the imagery, and how to enhance them, should be examined. Finally, whether increasing the experience of positive affect and positive expectations via imagery in everyday life can lead to significant reductions in anhedonia requires investigation.

While Theresa's report is anecdotal and should be seen as hypothesis‐generating, it is not unique: similar experiences have been reported by other participants in the trial (Blackwell et al., 2015) and in previous research (Blackwell & Holmes, 2010). More generally, Theresa's report highlights one potential benefit of examining individual cases in the early stages of developing an intervention because potentially informative data that is not captured by the standardised measures can otherwise be missed. Within the broad field of clinical CBM research, there is increasing recognition that the processes by which CBM interventions (and the control conditions commonly employed) may lead to symptom improvement are complex and multifaceted. Investigating examples of treatment success may help identify additional mechanisms of change and enable development of more powerful interventions (cf. Holmes et al., 2014).

### Implications for Treatment

The positive imagery intervention described in this article, here termed *positive imagery CBM*, is in the early stages of development. Further refinement of the paradigm and convincing demonstration of efficacy would be required before it could be recommended as a treatment in depression. However, Theresa's case highlights a number of points that are interesting in considering how positive imagery CBM could potentially be implemented as a treatment in future.

Theresa's case provides an illustration of a brief positive imagery CBM intervention apparently leading to striking benefits in the context of a complex presentation– in terms of not only depression severity and diagnostic comorbidity but also ongoing environmental stressors. While it is of course not possible to generalize from one case, such an illustration can provide a helpful counter‐example to challenge assumptions that might otherwise be made (Kazdin, 2014), for example, that such an intervention could only be helpful for mild cases, or that people experiencing severe symptom levels would not be able to engage with positive imagery or complete such a self‐directed intervention. In the face of such complexity, the temptation may be to attempt a complex intervention; however, it may be that focussing specifically on just one target or process, repeatedly and thoroughly, can sometimes be more helpful.

The worsening of Theresa's symptoms at the 6‐month follow‐up also illustrates potential limitations of a brief CBM intervention. Given her complex presentation and the ongoing environmental stressors, it would be surprising if a brief focussed intervention could have long‐lasting effects. While the format of a brief module delivered in isolation with follow‐up is convenient for research to isolate specific effects and find out how long they last, it is unlikely to represent how positive imagery CBM may best be deployed in a real world application. As Theresa herself remarked, the option to have “booster” courses if someone's mood started to dip again may provide a low‐intensity and scalable route to more sustained benefits.

Further, while Theresa's score on a measure of cognitive vulnerability, the SST, reduced over the course of the intervention, it remained high at posttreatment (more than 50% of sentences completed negatively, close to the pretreatment mean for participants in the trial), suggesting remaining cognitive vulnerability. Within the trial itself, SST scores posttreatment predicted levels of depression at the 6‐month follow‐up (Blackwell et al., 2015). In a real world application of a CBM intervention, it may be beneficial to persist with training until a dysfunctional bias has been adequately “trained out,” and the number of sessions required to do so will vary from one client to another.

Finally, it may be more realistic to see positive imagery CBM as an “add‐on” module to combine with another treatment (e.g. Internet‐delivered CBT; Williams et al., 2015) rather than a stand‐alone intervention. For example, positive imagery CBM could potentially offer a treatment adjunct specifically to increase positive affect and reduce anhedonia, given that these features of depression are often neglected in current treatment approaches and respond poorly (Dunn, 2012). If the “active ingredient” in the positive imagery CBM intervention is the generation of positive affect‐laden mental imagery, then the computerized paradigm as implemented in Theresa's case represents only one way of targeting this mechanism; incorporation of positive mental imagery into other interventions, whether computer‐delivered or otherwise, may increase their ability to enhance positive affect and reduce anhedonia. While treatments tend to target negative aspects of psychopathology associated with disorder onset and maintenance, enhancing positive aspects of experience may be particularly useful in promoting recovery (e.g. Teismann, Forkmann, Glaesmer, Egeri, & Margraf, 2016).

### Conclusions

The case presented illustrates the application of a computerized cognitive training intervention that involves repeated generation of positive mental imagery within the context of a complex clinical presentation. It highlights an interesting but not yet investigated mechanism (positive imagery “popping back” spontaneously to mind) that may contribute to symptom reduction over the course of treatment, and that would benefit from systematic investigation in future research. The experience of brief flashes of positive mental imagery in daily life may have a range of benefits in the context of depression, boosting mood, increasing positive expectations, and motivating behavior. Enhancing these potential effects of the training could hold particular promise for reducing anhedonic symptoms of depression. The development of simple computerized interventions to increase the experience of such positive mental imagery in daily life could provide a useful tool in the drive to improve treatment outcomes in depression.
